# Seedling Responses to Water Pulses in Shrubs with Contrasting Histories of Grassland Encroachment

**DOI:** 10.1371/journal.pone.0087278

**Published:** 2014-01-27

**Authors:** Steven R. Woods, Steven R. Archer, Susan Schwinning

**Affiliations:** 1 School of Natural Resources and the Environment, University of Arizona, Tucson, Arizona, United States of America; 2 Department of Biology, Texas State University, San Marcos, Texas, United States of America; Instituto de Biología Molecular y Celular de Plantas, Spain

## Abstract

Woody plant encroachment into grasslands has occurred worldwide, but it is unclear why some tree and shrub species have been markedly more successful than others. For example, *Prosopis velutina* has proliferated in many grasslands of the Sonoran Desert in North America over the past century, while other shrub species with similar growth form and life history, such as *Acacia greggii*, have not. We conducted a glasshouse experiment to assess whether differences in early seedling development could help explain why one species and not the other came to dominate many Sonoran Desert grasslands. We established eight watering treatments mimicking a range of natural precipitation patterns and harvested seedlings 16 or 17 days after germination. *A. greggii* had nearly 7 times more seed mass than *P. velutina*, but *P. velutina* emerged earlier (by 3.0±0.3 d) and grew faster (by 8.7±0.5 mg d^−1^). Shoot mass at harvest was higher in *A. greggii* (99±6 mg seedling^−1^) than in *P. velutina* (74±2 mg seedling^−1^), but there was no significant difference in root mass (54±3 and 49±2 mg seedling^−1^, respectively). Taproot elongation was differentially sensitive to water supply: under the highest initial watering pulse, taproots were 52±19 mm longer in *P. velutina* than in *A. greggii*. Enhanced taproot elongation under favorable rainfall conditions could give nascent *P. velutina* seedlings growth and survivorship advantages by helping reduce competition with grasses and maintain contact with soil water during drought. Conversely, *A. greggii*'s greater investment in mass per seed appeared to provide little return in early seedling growth. We suggest that such differences in recruitment traits and their sensitivities to environmental conditions may help explain ecological differences between species that are highly similar as adults and help identify pivotal drivers of shrub encroachment into grasslands.

## Introduction

**Figure 1:**
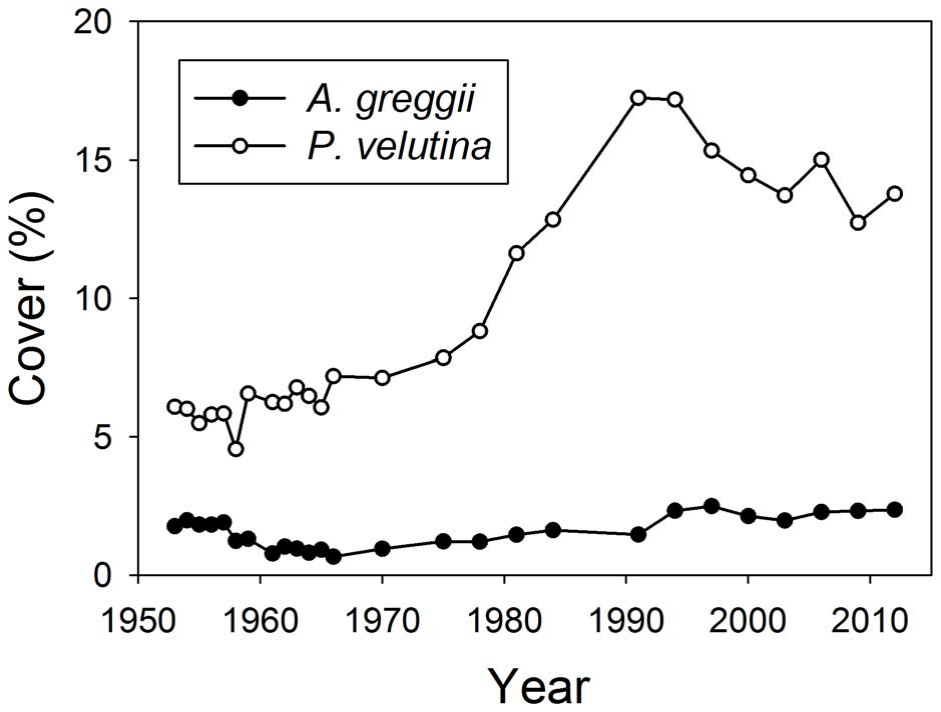
Canopy cover of *Acacia greggii* and *Prosopis velutina* shrubs in a grassland upland, Arizona, USA. Data were taken from the Santa Rita Experimental Range pasture 8 (from the Santa Rita Experimental Range Digital Database, http://ag.arizona.edu/SRER/longterm/ltcover.xls.). This pasture was grazed year-round by cattle at a stocking rate of 250–300 Animal Unit Months.

Encroachment of shrubs and trees in semiarid grasslands and savannas has been widely documented [1], [2]. In contrast to biological invasion, in which a non-native species is introduced and rapidly spreads through the community, woody encroachment often represents the increased abundance of shrub or tree species within or close to their historic ranges. To explain this phenomenon, studies of woody plant proliferation in grasslands have concentrated on the attributes of aggressively encroaching species, the grasslands they encroach into and the climatic and disturbance regimes that favor establishment [2]–[4]. However, one aspect often overlooked is that typically just one or very few of the many woody species in a regional flora encroach aggressively [5]. For instance, numerous shrub species are common in the Sonoran Desert of North America but, of these, only *Prosopis velutina* (velvet mesquite, a deciduous shrub) and *Larrea tridentata* (creosote bush, an evergreen) have spread rapidly and extensively into semi-arid grasslands of the region, under relatively mesic and xeric conditions, respectively [6]. Why is it that *P. velutina* has proliferated in grasslands while other native shrubs with similar growth form, function, life history and habitat, such as *Acacia greggii* (catclaw acacia) have changed little in abundance (Fig. 1)? We suggest that comparative studies [7] of encroachers and non-encroachers that co-occur in the same area may help identify key traits and drivers of woody encroachment, similar to approaches taken in invasion ecology [3], but with a focus on comparing native species.

Dryland shrub mortality rates are typically highest in the seedlings' first year [8], but woody plants are highly persistent once established. Bowers et al. [9] found approximately 25% mortality of long-lived perennials during the first 20 days after emergence in the Sonoran Desert, and Brown and Archer [10] found that most *Prosopis glandulosa* seedlings which survived the first two weeks survived at least two years. Among long-lived woody plants, it is therefore likely that differences in encroachment potential may be explained, at least in part, by differences in sensitivities to biotic and abiotic factors operating at the nascent stages of seedling establishment [11]. Attributes such as seed size, seedling growth rate, and patterns of above- vs. below-ground allocation may help predict what woody species are likely to be successful grassland invaders; but trade-offs among these traits are difficult to evaluate. For example, larger-seeded species typically produce bigger seedlings that have potential competitive and establishment advantages compared to smaller-seeded species [12], but smaller-seeded species tend to have shorter emergence times and higher seedling growth rates compared to larger-seeded species [13]. Rapid emergence may be particularly beneficial where conditions suitable for establishment are ephemeral, a common characteristic in arid and semi-arid environments [14].

Successful establishment of woody species in water-limited, grass-dominated ecosystems has also been linked to the rapid development of a taproot that can provide the seedling with access to persistent water sources at depths not effectively exploited by grasses [15], [16]. This is particularly important for woody species that recruit in the warm season when the availability of near-surface soil moisture can be highly episodic [17]. In arid and semi-arid regions of North America, a great majority of rainfall events supply less than 10 mm water [18]. Some Sonoran Desert perennial grasses can germinate with 11 mm rainfall or less [19], [20]. However, shrub and tree seeds in the region typically germinate and emerge as seedlings after rainfall events in excess of 15 mm [9], [21]. A single germination-triggering rainfall event of 15–20 mm may keep the top 20 cm of soil hydrated for up to 20 days [22], but beyond this period a woody seedling can be assured of continual water supply only if water infiltrates deeper and roots extend below the dry zone. A succession of large rainfall events can produce deep percolation that permits taproot extension and favorable establishment conditions [23], but this happens in a minority of years in arid and semi-arid regions [24]. Interactions between precipitation pulses and early seedling development vary between species and may help explain differences in recruitment dynamics among woody species in drylands [25].

This study sought to evaluate the influence of water supply patterns on early seedling development in an aggressive grassland encroacher (*P. velutina*) and a co-occurring non-encroacher (*A. greggii*), and the extent to which these species differ in seed size, germination dynamics, growth rate, taproot development and root and shoot mass. Both species are common in riparian areas [6], but *P. velutina* has markedly proliferated in uplands in recent decades (Fig 1). Furthermore, while both have been introduced outside of North America, *P. velutina* has become invasive in Australia [26] and southern Africa [27], whereas *A. greggii* has not [28].

Can differences in shrub seed germination/seedling development attributes help explain these differences in grassland encroachment potential? We hypothesized that seedlings of the aggressive encroacher, *P. velutina*, would exhibit greater responsiveness to water supply than the non-encroacher, *A. greggii*. More specifically, we hypothesized that the aggressive encroacher would have (1) earlier emergence, (2) higher growth rate and (3) greater taproot elongation. These hypotheses were tested by germinating seeds and growing seedlings in a glasshouse for 16–17 days under watering regimes simulating a wide range of rainfall conditions. We chose this short experimental duration because the days and weeks following germination are often the most critical for recruitment [8]–[11] and to minimize artificialities associated with root development in pots. An experiment of this duration was expected to reveal characteristic differences between species related to their abilities to develop deep tap roots and, by extension, their survival odds, if such differences existed.

## Materials and Methods

### Experimental species

*Prosopis velutina* Woot. (velvet mesquite) and *Acacia greggii* A. Gray (catclaw acacia) are members of the Fabaceae and native to the Sonoran Desert of northern Mexico and southwestern USA [29], [30]. They are long-lived shrubs with arborescent growth potential [6]. Both species are winter-deciduous and drought tolerant [6], [31], can regenerate vegetatively after fire [32], and are deeply rooted when mature [33], with roots reportedly capable of exceeding 50 m depth [34], [35].

We obtained the seeds used in our experiment from Desert Seed Source, Tempe, Arizona. *A. greggii* seeds were from the vicinity of Oracle, Arizona and *P. velutina* seeds were from nearby Sonora, Mexico. Although we could not control for seed age *per se*, seeds of both species are known to remain viable decades [36] and were collected within 3 y of the study.

### Experimental design and context

We conducted the study in June and July 2006 in a glasshouse at the University of Arizona Campus Agricultural Center in Tucson, Arizona, USA on potted plants subjected to eight different watering regimes. Each species×water combination was replicated 12 times and randomly assigned within three blocks arranged by distance from an evaporative cooler. Species, watering treatments and soil type were chosen to represent conditions at the Santa Rita Experimental Range (SRER), Arizona, USA (32°16′N, 110°56′W), a Sonoran Desert field site with long-term records of climate and vegetation dynamics which has undergone significant woody plant encroachment.

*P. velutina* has proliferated markedly at SRER since 1902, such that *P. velutina* presently dominates what were historically grasslands on sandy loam or loamy sand soils in the 900–1300 m elevation zone [37], [38]. Other woody members of the Fabaceae present at lower densities than *P. velutina* include *A. greggii* and *Parkinsonia florida* (Benth. ex A. Gray) S. Watson. *Larrea tridentata* (Sesse & Moc.) Cov. (creosote bush) is the dominant shrub at lower, drier elevations and *Quercus* spp. dominate at higher elevations. Mean annual precipitation at SRER increases with altitude, ranging approximately from 275 mm at about 900 m elevation to 450 mm at 1400 m elevation, and occurs predominantly in the summer monsoon with a smaller peak in winter [37].

Watering treatments bracketed precipitation levels considered sufficient to trigger germination and likely to permit establishment in the Sonoran Desert. In this region, precipitation events generally have to be at least 15 mm to result in significant germination of woody plant species [9]. At SRER, mean daily precipitation during the peak of the summer monsoon season (July–August) averages 2.6 mm d^−1^. Large events delivering approximately 10 mm d^−1^ over 5 consecutive days are relatively rare, occurring on average once every 3 yr. Events of shorter duration, e.g. those that deliver 15 mm over 2 days, on average occur 7 times yr^−1^. We sought to simulate a representative range of these precipitation conditions by imposing a factorial watering treatment with variable intensities of germination triggering events (the ‘pulse’ treatment, 10 mm per day for the initial 2, 3, 4 or 5 days) and varying the subsequent watering frequency (the ‘maintenance’ treatment, 5 mm either daily or on alternate days). This resulted in eight total watering treatment combinations which supplied between 55 and 100 mm of water over a 16 or 17 day period (Table 1).

**Table 1:** Total amount of water applied under watering treatments.

Maintenance regime:	Pulse duration: initial number of consecutive days at 10 mm d^−1^			
5 mm on alternate or all days following the initial watering pulse	2	3	4	5
Alternate	55 mm	60 mm	70 mm	75 mm
Daily	90 mm	90 mm	100 mm	100 mm

Watering treatments were initial pulse duration (four levels, from 2 to 5 days with 10 mm water per day) and subsequent maintenance regime (two levels, applying with 5 mm water either every day or on alternate days) over a combined duration of 16 or 17 days. Treatments were based on long-term precipitation records at the Santa Rita Experimental Range, Tucson, AZ, USA.

We filled the pots (7.6 cm square by 35.6 cm deep) with sandy loam soil from the SRER. Gravimetric soil N and organic C content were 0.11±0.01‰ and 1.07±0.03‰, respectively, carbonate C was 5.85±0.17‰ and gravimetric water content at field capacity was 16.8±1.6%. Maximum plant dry mass per pot volume was 0.16 g L^−1^, an order of magnitude lower than the 2 g L^−1^ upper limit recommended by Poorter et al. [39] to avoid pot size effects on growth.

In our experiment, all water that was applied infiltrated within a few seconds. This is not necessarily the case under all field conditions. For example, biological soil crusts can decrease surface infiltration rate and amount, though in some cases they can have opposite effects [40]. The potted soils in our study had no crust, which is typical for Sonoran Desert grasslands, where biological crusts are at best early successional and dominated by cyanobacteria, but more commonly absent at sites frequently disturbed by grazing, as is the case at the SRER [41]. Infiltration depths are also influenced by soil bulk density [42]. Bulk density of the soils in our experiment was 1.43±0.06 g cm^−3^ which is within the range of values reported for SRER soils of similar texture and pedogenesis [43]–[45]. The infiltration characteristics in our trials are therefore expected to have been comparable to field conditions at the SRER.

We closed pots off at the bottom with weed barrier cloth to prevent soils loss. Over the short duration of the experiment there was no water leakage from the pots, and taproots did not reach the bottom of the pots in any of the watering regimes.

### Experimental methods

Randomly selected air dry seeds of each species were weighed individually (n = 30 per species). We then removed seed coats manually and reweighed the seeds. Seed coat dormancy was overcome using recommended chemical scarification techniques [36], [46] [*P. velutina*: 10 min in 20% H_2_SO_4_(aq.), *A. greggii*: 20 min in 80% H_2_SO_4_(aq.)]. However, interpretation of our recruitment data is predicated on the unverified assumption that these treatments did not differentially alter time to emergence. In the Fabaceae, time to germination has been reported to increase [47], decrease [48]–[50], vary non-systematically [51] or be independent of [52] duration or concentration of acid scarification. However, of these studies, those reporting significant effects failed to control adequately for experiment-wise Type I error rates in multiple comparisons [53], [54]. In addition, Zare et al. [50] found two durations of sulphuric acid scarification had no effect on time to emergence in *Prosopis juliflora*, which, depending on the variety studied, is either closely related to or synonymous with *P. velutina* [30].

We imposed the watering regime on day 1 of the experiment at the level of individual pots through a drip irrigation system. Scarified seeds were soaked in water for 24 h in the dark immediately prior to planting on day 2. Every pot was planted with 4 seeds at a depth of approximately 10 mm.

Mean emergence was 78%±3% for *P. velutina* and 62%±3% for *A. greggii*. We recorded day of first emergence for all pots. We also recorded the emergence day of second and later emerging seedlings per pot, but then removed them to prevent competition. We staggered the start of the experiment over five days so that the maintenance watering regime could be established on the same day. Seedling harvests were also staggered, occurring either on day 16 (for 3 and 5 pulse days) or on day 17 (for 2 and 4 pulse days) of the experiment, with the purpose of allowing one day without watering immediately before the harvest in all treatments. At harvest, we separated roots from the soil manually and measured taproot length immediately. We separated roots from shoots and oven dried both at 70°C for 48 h prior to weighing them. Results are presented in terms of days since imbibition rather than days since emergence since the former is initiated in response to a precipitation event(s) and subsequent seedling survival would depend on (among other things) the time from imbibition to the next water pulse.

### Data analysis

We compared seed mass, with or without seed coat, between *P. velutina* and *A. greggii* with Student's t tests assuming unequal variances. Preliminary analyses indicated no significant effect of block on seedlings (all *P*>0.05), so we omit it from all analyses reported here.

We performed ANOVAs according to the general model:
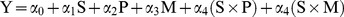
(1)where Y is the dependent variable, and independent variables included species (S, 2 levels), pulse duration (P, 4 levels) and maintenance watering regime (M, 2 levels). Dependent variables in ANOVAs were time to emergence (the number of days from seed imbibition to the first appearance of cotyledons above the soil surface); absolute growth rate (AGR); root, shoot and total dry mass; and taproot length.

AGR was calculated as follows [55]:
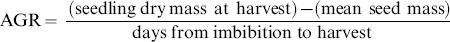
(2)where the mean seed mass was measured exclusive of the seed coat (mean ± S.E.M.: 22±1 and 196±11 mg for *P. velutina* and *A. greggii*, respectively; n = 30 for each species). We used AGR in preference to relative growth rate to avoid confounding growth rate differences with seed mass differences [56].

Time to emergence varied widely among seedlings, giving seedlings different amounts of time for photosynthesis. To assess the effect of time to emergence on AGR, root, shoot and total biomass, we conducted ANCOVAs according to the model:
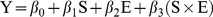
(3)where E is days to emergence of harvested seedlings, i.e. the first seedling to emerge per pot, and Y is the dependent variable.

The relationship between taproot length and pulse duration (2–5 days) was approximately linear, so we used linear regression analysis in a mixed model to quantify the proportional effect of pulse duration on taproot growth. The model was as in equation (1), except that P was treated as a continuous rather than ordinal variable.

We used the Tukey-Kramer test to determine differences in multiple comparisons except for differences between species' overall means and slopes with respect to days to emergence, for which Student's t tests were used. Where necessary, log transformations were used to meet test assumptions of normality and equal variance. We performed all analyses using JMP 9.0 (SAS Institute, Inc., Cary, NC, USA). All results are reported on a per live seedling basis.

## Results

### Seed mass

The mass of *P. velutina* and *A. greggii* seeds used in our study fell within the ranges reported in other studies of these species [57], [58]. Mean (± S.E.M.) *A. greggii* total seed mass (237±10 mg) was 7-fold greater than that of *P. velutina* (35±2 mg) (*t* = 20.45, df = 30·92, *P*<0.0001). Mean mass with seed coats excluded was 196±11 and 22±1 mg, respectively, a 9-fold differential (*t* = 15.18, df = 29.71, *P*<0.0001).

### Time to emergence

**Figure 2:**
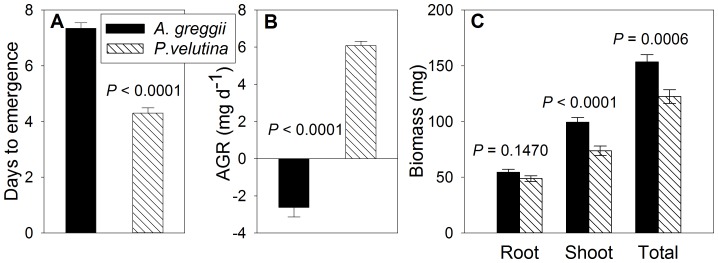
Mean time to emergence, absolute growth rate (AGR) and dry biomass in young shrub seedlings. Species were *Prosopis velutina* (n = 96) and *Acacia greggii* (n = 91). Time to emergence was evaluated by within-pot averages from 4 seeds pot^−1^. Biomass was measured 16 and 17 days after imbibition. AGR was calculated according to equation 2, excluding seed coat mass. *P* values are from *t* tests.

Mean (± S.E.M.) days to seedling emergence for *P. velutina* and *A. greggii* were 4.3 (±0.2) and 7.3 (±0.2), respectively. Thus, *P. velutina* had potentially 3 more days on average for carbon assimilation (Fig. 2a, Table 2). Species×watering regime (pulse days, post-pulse frequency) interactions were not significant.

**Table 2:** ANOVA analyses for days to emergence, absolute growth rate (AGR; eq. 2), total seedling dry mass and taproot length.

Treatment	DF	Log (Days to Emergence)			AGR			Total Mass			Taproot Length		
		%SS	F-ratio	*P*	%SS	F-ratio	*P*	%SS	F-ratio	*P*	%SS	F-ratio	*P*
Model	9	50	19.74	<0.0001	66	36.66	<0.0001	22	5.34	<0.0001	46	16.92	<0.0001
Species	1	42	147.63	<0.0001	44	72.43	<0.0001	6	12.33	0.0006	1	3.35	0.0690
Pulse days	3	7	7.75	<0.0001	8	4.23	0.0065	6	4.68	0.0036	33	36.33	<0.0001
Post-pulse frequency	1	0	1.48	0.2255	2	2.64	0.1059	1	2.75	0.0991	8	27.90	<0.0001
Species×Pulse days	3	2	2.39	0.0706	9	4.90	0.0027	5	3.68	0.0133	2	2.45	0.0655
Species×Frequency	1	0	0.00	0.9792	4	6.45	0.0120	3	6.81	0.0099	0	0.61	0.4348

Days to emergence was analysed for within-pot averages (4 seeds pot^−1^). AGR, total mass and taproot length were evaluated on the first seedling to emerge per pot. Watering treatments were pulse duration (number of days at start of experiment with 10 mm water per day) and follow-up watering frequency (post-pulse frequency of 5 mm watering events). Seedlings of *P. velutina* (n = 96) and *A. greggii* (n = 91) were thinned as needed to one per pot and harvested 16 and 17 days after imbibition was initiated.

### AGR and Biomass

There was no seedling mortality over the course of the experiment. Across watering treatments, AGR (equation 2, seed coat mass excluded) was higher in *P. velutina* than in *A. greggii* (6.1±0.2 versus −2.6±0.5 mg d^−1^, respectively; Fig. 2b, Table 2), but whole-plant biomass at harvest was lower in *P. velutina* than in *A. greggii* (122±4 versus 153±8 mg, respectively; Fig. 2c; see Table 2). This reflected primarily a difference in shoot mass (74±2 mg in *P. velutina* versus 99±6 mg in *A. greggii*), as root mass was not significantly different (Fig. 2c). Root/total mass also did not differ significantly between *P. velutina* and *A. greggii* seedlings (0.40±0.02 and 0.35±0.03, respectively).

**Figure 3:**
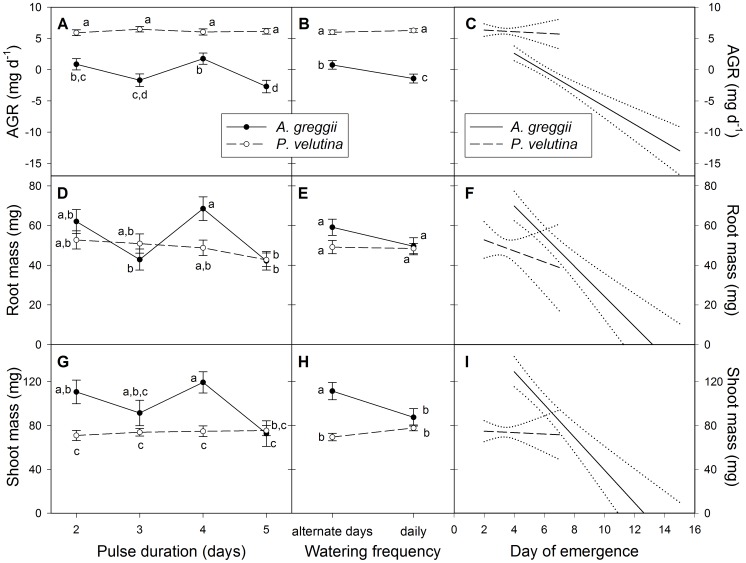
Growth rate and biomass responses of young shrub seedlings to watering treatments. Mean (± S.E.M.) AGR (equation 2; a, b, c) and oven-dry root mass (d, e, f) and shoot mass (g, h, i) of *Prosopis velutina* (n = 96) and *Acacia greggii* (n = 91) seedlings 16 and 17 days after imbibition, in response to pulse duration (number of days at start of experiment with 10 mm water per day; panels a, d and g) and follow-up watering (post-pulse frequency of 5 mm watering events; panels b, e and h). Panels c, f, and i depict regressions against day of emergence (time between imbibition and cotyledon emergence) of harvested seedlings for *P. velutina* (dashed lines) and *A. greggii* (solid lines). Means with different letters were significantly different (Tukey-Kramer test, **α** = 0.05).

In *A. greggii*, AGR and root, shoot and total mass varied significantly, albeit without consistent pattern, across watering treatments, whereas *P. velutina* was relatively insensitive to watering levels (Fig. 3). This variation was highly correlated with time to emergence in *A. greggii* (all *P*<0.0001), but not in *P. velutina* (all *P*>0.30; Fig. 3c,f,i; Table 3). Overall, species×watering regime interactions were significant (Table 3).

**Table 3:** Summary of ANCOVA analyses for absolute growth rate (AGR, eq. 2) and dry biomass of *P. velutina* (n = 96) and *A. greggii* (n = 91).

Treatment	DF	AGR			Root mass			Shoot Mass			Total Mass		
		%SS	F-ratio	*P*	%SS	F-ratio	*P*	%SS	F-ratio	*P*	%SS	F-ratio	*P*
Model	3	70	137.22	<0.0001	19	14.66	<0.0001	33	29.83	<0.0001	31	26.89	<0.0001
Species	1	56	69.67	<0.0001	10	13.19	0.0004	22	30.36	<0.0001	19	27.08	<0.0001
Day of emergence	1	8	10.39	0.0015	8	9.72	0.0021	7	9.26	0.0027	8	11.09	0.0011
Species×day of emergence	1	5	6.77	0.0100	2	2.00	0.1589	5	7.31	0.0075	4	5.76	0.0175

Seedlings were harvested 16 and 17 days after imbibition was initiated.

### Taproot development

**Figure 4:**
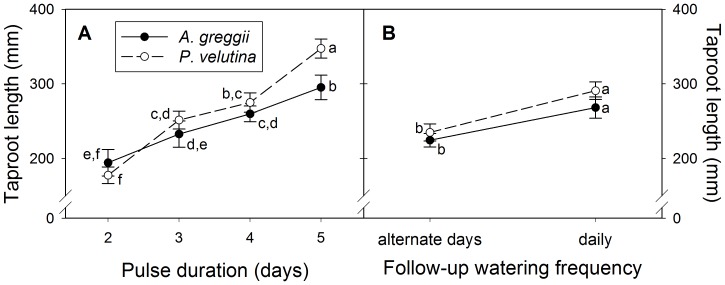
Taproot length responses of young shrub seedlings to watering treatments. Mean (±S.E.M.) taproot length of *Prosopis velutina* (n = 96) and *Acacia greggii* (n = 91) seedlings at harvest (16 and 17 days post-imbibition) in response to pulse duration (number of days at start of experiment with 10 mm water per day) and follow-up watering (post-pulse frequency of 5 mm watering events). Values with different letters differed significantly (Tukey-Kramer test, **α** = 0.05).

Taproot length increased with pulse duration and with maintenance watering frequency, and the response was similar between species (Fig. 4, Table 3). Within treatments, significant species differences were observed only for the longest pulse duration (5 days) and for this treatment, *P. velutina* had longer taproots than *A. greggii* (348±13 mm vs. 295±16 mm; Fig. 4a).

The regressions of taproot length on pulse duration had significantly different slopes across species (*t* = 2.54, df = 1, *P* = 0.0121; mixed model treating pulse duration as continuous, equation 1). Each 10 mm of water added during the pulse phase increased taproot length by 53±6 mm in *P. velutina* and just 32±6 mm in *A. greggii* (both *P*<0.0001). Thus, the response to pulse duration was 65%±28% greater in *P. velutina* than in *A. greggii*.

Doubling the water added during the maintenance phase from, on average, 30 mm to 60 mm (Table 1) increased taproot length 56±13 mm in *P. velutina* and 42±13 mm in *A. greggii* (*t* = 4.37, df = 1, *P*<0.0001 and *t* = 3.14, df = 1, *P* = 0.0020, respectively). Thus, for both species, the effect of an additional 10 mm water during the initial pulse was comparable to the average effect of an additional 30 mm water during the subsequent maintenance phase.

## Discussion

This study sought to determine whether an explanation for why some shrubs have recently increased in abundance in grasslands, whereas others have not (Fig. 1), might be related to patterns of germination and early seedling growth in response to soil moisture availability. Our glasshouse pot experiment enabled us to tightly constrain environmental conditions and examine intrinsic responses of *P velutina* and *A. greggii* seedlings to variation in watering regimes while controlling for other factors that might mask those fundamental differences. An important next step would be to investigate how the intrinsic growth differences identified in our experiment play out in a natural context where competition, herbivory, and disturbance (e.g., fire) occur.

*P. velutina*, the aggressive woody encroacher, had smaller seeds but emerged earlier, had a higher growth rate and, despite allocating a similar proportion of total biomass to roots, had taproot elongation rates that were more responsive to the amount of initial water application compared to the non-encroacher, *A. greggii*. The similarity in species' seedling mass at the end of the experiment is remarkable, considering that *P. velutina* started with an embryonic mass only 11% that of *A. greggii*. *P. velutina* was able to close the size gap quickly, increasing its biomass nearly 6-fold compared to its seed mass, while over the same period *A. greggii* exhibited slight net negative growth relative to its initial seed mass (Fig. 2b). This suggests that *P. velutina* started autotrophic growth earlier than *A. greggii* or had cotyledons/true leaves with higher photosynthetic capacities.

The study showed that in a semi-arid environment, where favorable conditions for seedling growth are short-lived owing to the preponderance of small, pulsed rainfall events, greater seed mass does not necessarily confer seedling size or establishment advantages, if it is associated with longer emergence times. In the larger-seeded *A. greggii*, AGR was strongly negatively correlated with days to emergence, i.e., individuals that emerged later were smaller at harvest. Evaluating the regression of AGR against days to emergence (Fig. 3c) at day 4 (the average number of days that *P. velutina* took to emerge) suggests that *A. greggii* could have accumulated biomass almost as fast as *P. velutina*, had it emerged at the same time. Thus, apart from differences in time to emergence, there was little difference in growth potential between the two species.

The relationship between seed mass and time to germination varies widely among species [59] but among trees, small-seeded pioneer species tend to germinate more rapidly than large-seeded late seral species [60]. The greater seed mass in *A. greggii* compared to *P. velutina* may reflect an adaptation in the former to recruiting in more competitive, wooded habitats such as those in riparian zones, with *P. velutina* being better adapted to seizing brief recruitment opportunities created by disturbance in more open and less competitive environments [12].

Seedling biomass accumulation was not limited by water supply in this experiment, but water supply patterns still had large effects on seedling development, particularly on taproot extension. This is important, because access to deeper, persistent soil moisture is critical to survivorship past the emergence and early growth phase and to establishing seedlings with some degree of resilience to rainfall fluctuations. For example, between rooting depths of 16 cm and 35 cm in a semi-arid environment, woody seedling survival increased approximately linearly both with rooting depth and with moisture at the deepest soil reached by roots [16], and in an arid environment, shrub seedling survival through the wet season increased with water supply, and survival to one year occurred only in a species which reached relatively stable soil moisture at 40 cm depth within 7 months of germination [25]. This suggests that day-by-day variation in precipitation patterns over the short period of seedling emergence and the first days of seedling growth can have disproportionally large effects on shrub recruitment in a given year. In this context, the slower taproot elongation of *A. greggii* under the largest watering pulse may have negative consequences on seedling survivorship in a field setting. At the highest water supply rate, taproots in *A. greggii* were more than 5 cm shorter than those of *P. velutina*, suggesting significantly less soil depth access to roots within the 40 cm top-soil layer in which most soil water is available [61], [62]. In comparison, taproots of *P. velutina* seedlings in the field reached 38 cm depth at the end of their first growing season and 69 cm after their second [63], and seedlings of the related species *P. glandulosa* had taproots extending to 20 cm with cotyledons present and one true leaf, reached depths >40 cm four months after germination, and after one year depended primarily on soil moisture below 30 cm [15].

The taproot elongation response to initial water pulse duration in our study was near-linear and, compared by units of water applied, stronger than for water applied in smaller doses in the subsequent maintenance phase. This is explicable by the effect of pulse regime on water percolation depth and inhibition of root elongation in the transition zone from wet to dry soil at the wetting front. Root elongation is halved by matric potentials lower than about −0·5 MP in some species, so roots are generally prevented from growing into dry soil [64]. In our experiment, repeated initial applications of 10 mm water on consecutive days would have driven the wetting front deeper into the soil column by fast, near-saturated flow [65] and in turn would have allowed taproots to grow deeper. Once the pulse regime stopped, water would have infiltrated further down more slowly by sub-saturated flow, explaining why water applied during the maintenance phase had less of an effect on taproot elongation. Informal observations at harvest were consistent with this interpretation (e.g., soil appeared and felt moist from top to bottom in the highest pulse treatment, and was moister at the top and drier towards the bottom in the lowest pulse treatment).

### Implications for woody plant encroachment into grasslands

The observation that taproot development was regulated almost independently of root biomass creates additional complexity for dynamic savanna models and the prediction of woody encroachment trends with climate change. For example, a recent study identified the “window of opportunity” for recruitment in *P. velutina* as a summer with non-limiting precipitation [4]. Our experiment indicates that the definition of “non-limiting” precipitation is not straightforward, as optimal taproot development during the critical first few weeks of a seedling's life may require far more precipitation than is necessary to maximize biomass accumulation *per se* during later weeks and months of a seedling's first year. Thus, total seasonal precipitation is likely to be an overly coarse measure for assessing establishment opportunities [66], and recruitment models may be improved by also incorporating effects of above-average precipitation events on depth of water penetration into the soil, seedling rooting depth, and subsequent survival.

Shrub or tree recruitment in arid and semi-arid environments is often thought to be “episodic”, but there is an ongoing debate whether savanna dynamics or shrub encroachment are a consequence of intermittent recruitment pulses or relatively continuous but low recruitment rates [9], [11], [67], [68]. Models suggest both may be required to maintain populations or explain invasive success [69], [70], but numerical analyses beg the biological question: if seedlings can grow and survive on average amounts of precipitation (continuous establishment model), what additional advantages are conveyed in very high rainfall years that could cause recruitment spikes? The interaction between water percolation depth and taproot development could be the answer. Strong rainfall events at critical times may be associated with extraordinarily rapid elongation of taproots in those species that prioritize the development of a deep root system. This could have non-linear, positive effects, if not on growth rate then on survival in subsequent drought periods. Conversely, it is possible that weaker taproot response may cause a lack of distinct *A. greggii* recruitment spikes in Sonoran Desert grasslands (Fig. 1). If so, this could partly explain the different encroachment histories of *P. velutina* and *A. greggii*.

Differences in *P. velutina* vs. A. *greggii* seedling establishment potential in grasslands may be magnified by additional life-history contrasts, such as differences in dispersal potential. Seeds of *P. velutina*, and its close relative *P. glandulosa*, are encased in fleshy pods high in energy and nutrients [71]. These pods are consumed by domestic livestock (cattle, sheep and horses), and a high proportion of the seeds ingested with the pods can escape mastication and be widely dispersed [72]. Livestock are highly effective agents of *Prosopis* seed dispersal not only because they disperse large numbers of seeds away from parent plants harboring seed predators, but because they also facilitate germination by scarifying hard seed coats, and enhance seedling establishment by depositing viable seed in grazed areas where grass interference and the probability of fire have been reduced [73]. In contrast, seeds of *A. greggii* are contained in dry, leathery pods that are unpalatable to livestock [31], [74]. Therefore, while *A. greggii* may dominate or co-dominate arroyos and washes where flash flooding disperses seeds and scarifies them [75], its opportunities for dispersal into uplands may be more limited than those of *P. velutina*. Thus, differences in seed dispersal coupled with differences in seedling establishment potential may help explain why two species representing ostensibly similar plant functional groups differ so markedly in their abilities to invade grasslands (Fig. 1).

Woody plant establishment in drylands may be affected by herbivory, herbaceous competition and fire as well as by seed availability and soil moisture [76]. Herbivory can be a significant cause of woody seedling mortality [77] and removal of seed and seedling predators can promote shrub recruitment [78]. Herbaceous competition can reduce woody plant establishment but is often insufficient to exclude woody plants from grasslands [76], and competitive effects of grasses on young shrub seedlings can be low even on sites with high levels of grass cover [10], [15], [67]. Moreover, microsites with low levels of herbaceous cover and competition are often numerous in semi-arid grasslands [79] and woody plants often establish during periods of relatively high soil moisture, when below-ground competition from grasses is reduced [76]. However, grasses can fuel fires which can suppress or kill woody plant seedlings and saplings, and thereby prevent encroachment into grasslands [76], [77]. Thus, while taproot elongation may be necessary for woody seedling recruitment in grassland, it may not be sufficient in and of itself unless constraints imposed by dispersal, fire, herbivory and/or competition are also relaxed or overcome.

While multiple factors are likely to influence woody plant population dynamics, there is broad consensus that the worldwide phenomenon of woody encroachment has been triggered, at least initially, by a release from recruitment limitations accompanying changes in land use and/or climate [1]. This implies that the critical differences between species that did and did not became woody encroachers involved traits that (a) were critical to reproduction (e.g., seed production, dispersal, dormancy, germination) and seedling growth (biomass allocation and physical structure); (b) were sensitive to changes in climate, atmospheric CO_2_ or disturbance regimes; and (c) would have positioned woody encroachers to achieve greater recruitment success in the new setting. Some of these regime changes are unlikely to have distinguished between *A. greggii* and *P. velutina* (e.g., historically more frequent grassland fires would have suppressed both species) and others may have favored *A. greggii* more than *P. velutina*, rather than vice versa. We suggest that an evaluation of seed and seedling traits critical to early survival, and their differential sensitivities to historic regime changes, may help explain not just why some woody species were better able to encroach into grasslands than others, but also which of the multiple possible drivers of change were pivotal.
